# Vascular decellularization dynamics: Optimizing Triton X-100 and SDS concentration with incubation time

**DOI:** 10.34172/jcvtr.33164

**Published:** 2024-09-20

**Authors:** Umer Bin Shahzad, Ume Aiman, Muhammad Ahmed Sheikh

**Affiliations:** Islamic International Medical College, Rawalpindi, Pakistan

## Dear Editor,

 I am writing to discuss a riveting aspect of the study “Acellular carotid scaffold and evaluation the biological and biomechanical properties for tissue engineering” by Farina Rashidi et al.^1^ With coronary artery disease (CAD) affecting 154 million people and peripheral artery disease (PAD) impacting 120 million globally, representing 32.7% and 25.6% of the cardiovascular disease burden respectively, there is an urgent need for new strategies to address this growing health. In this case, acellular scaffolds produced by decellularizing allogeneic or xenogeneic arteries seem like suitable replacements. Decellularization of allogenic or xenogeneic vascular scaffolds, preserves extracellular matrix (ECM) components and mimics blood vessel geometry, has garnered significant interest.^2^ However, an established decellularization protocol with the perfect equilibrium of concentration and incubation duration has yet to be defined.

 The study by Farina Rashidi et al notably highlights the efficacy of Triton X-100 and tri(n-butyl) phosphate (TnBP) in the decellularization process of carotid arteries.^1^ The utilization of 1% Triton X-100 and 1% TnBP yielded decellularized vessels devoid of residual cell nuclei and cellular components, with well-preserved collagen fibers and a significant reduction in DNA content to less than 50 ng/mg of dried tissue (*P* < 0.001). Additionally, there were no notable alterations in the chemical composition of the ECM, indicating the successful integration of mechanical properties in the decellularized vessels. ^1^ However, the key determinant of decellularization efficacy lies in selecting the appropriate concentration of reagents and duration of incubation.

 Although the study by Farina Rashidi et al provide valuable insight into the potential of Triton X-100 in efficiently decellularizing the blood vessels, any combination of detergents leads to structural damage to the intrinsic ECM, with the extent of damage escalating alongside increased detergent concentration. The debate remains contentious over whether to increase the concentration of chemical agents, which would reduce reagent quantity and cost but potentially cause greater ECM damage, or to extend the decellularization time, which could affect the rate of decellularization. The most effective approach lies in balancing the time and concentration of reagents to achieve optimal outcomes.^3^ An optimal decellularization regimen involving 0.5% Triton X-100 for 24 hours coupled with 0.25% SDS for 72 hours presents an optimal approach, yielding a vascular ECM and three-dimensional structure with minimal disruption.^4^ Furthermore, several investigations on the concentration of reagents to be used yielded similar outcomes, showcasing that the combination of 0.25% sodium dodecyl sulfate (SDS) and 0.5% Triton X-100 emerged as an optimal decellularization protocol, effectively eliminating cells while preserving the integrity of the extracellular matrix. ^5^

 In conclusion, Farina Rashidi et al demonstrate that Triton X-100 effectively decellularizes carotid arteries, preserving collagen fibers and significantly reducing DNA content offering potential in CAD and peripheral vascular diseases. Optimal reagent concentration and incubation time are crucial to minimize ECM damage. We recommend the combination of 0.5% Triton X-100 for 24 hours and 0.25% SDS for 72 hours as recommended by various researched.

## Competing Interests

 The author declares no conflict of interest in this study.

## Ethical Approval

 Not applicable.
